# ﻿First report of albinism for *Achalinussheni* (Serpentes, Xenodermidae), with extended diagnosis of the species

**DOI:** 10.3897/zookeys.1209.128944

**Published:** 2024-08-06

**Authors:** Yu-Hao Xu, Shuai Wang, Shun Ma, Frank T. Burbrink, Li-Fang Peng, Song Huang

**Affiliations:** 1 State Key Laboratory of Plateau Ecology and Agriculture, Qinghai University, Xining 810016, China Qinghai University Xining China; 2 Chengdu Institute of Biology, Chinese Academy of Sciences, Chengdu 610041, China Chengdu Institute of Biology, Chinese Academy of Sciences Chengdu China; 3 University of Chinese Academy of Science, Beijing 100049, China University of Chinese Academy of Science Beijing China; 4 Department of Herpetology, The American Museum of Natural History, New York NY 10024, USA Department of Herpetology, The American Museum of Natural History New York United States of America; 5 Anhui Province Key Laboratory of the Conservation and Exploitation of Biological Resource, College of Life Sciences, Anhui Normal University, Wuhu 241000, China Anhui Normal University Wuhu China

**Keywords:** Hunan Province, morphological characters, phylogeny, Shen’s Odd-scale Snake, Xenodermidae

## Abstract

Albinism is an uncommon phenomenon and inherited condition in animals characterized by a partial or complete lack of melanin. The family Xenodermidae Gray, 1849, is a group of caenophidian snakes widely distributed in South, East, and Southeast Asia, including five recognized genera and 36 species. However, there are currently no reports of albinism in any species in Xenodermidae. *Achalinussheni* Ma, Xu, Qi, Wang, Tang, Huang & Jiang, 2023 was first described based on five male specimens from Loudi City and Nanyue District, Hunan Province, China. At the time, there were no descriptions on female individuals. In this study, we report in detail a collected albinistic specimen of *A.sheni*, which is the first discovery of wild albinism in the family Xenodermidae. We also provide photographs and descriptions of the first three female specimens of *A.sheni* and extend the diagnosis of this species.

## ﻿Introduction

Coloration in most organisms evolved to respond to the background environment (Bechtel 1978; Krecsák 2008; Bruni 2017; Lu et al. 2024; Sun et al. 2024), and melanocytes that function to produce and store melanin play a crucial role in physiological color adaptations. However, the variation of melanocytes can also lead to corresponding chromatic anomalies, like the occurrence of albinism, which is usually characterized by a partial or complete lack of melanin. This condition is usually has been the result of tyrosinase inactivation caused by autosomal recessive mutations (Griffiths et al. 1998; Alberts et al. 2004; Krecsák 2008; Abegg et al. 2015).

In squamate reptiles, albinism is one of the most striking aberrations of body color pattern, which is usually divided into two types: 1) complete albinism showing the complete absence of melanin in the entire body with the red eyes and a pastel yellow, yellow, or white body coloration; and 2) partial albinism manifested as reduction in melanism across the whole body, rather than complete disappearance, resulting in a lighter body coloration (Sazima and Pombal 1986; Sazima and Di-Bernardo 1991; Hoshing and Mahabal 2013; Abegg et al. 2015). Often under artificial conditions, albino reptiles have been well documented, and many species with albino variants have been bred in large numbers for the pet market (Bechtel 1991; Bechtel 1995; Broghammer 2000). However, the wild albino phenomenon is usually extremely rare, due to the high probability of stillborns or malformations, such as linked anatomical abnormalities affecting eyesight, communications, and sexual selection, difficulty thermoregulating, and easy detection by prey and predators (Roulin and Bize 2007; Dutta et al. 2022).

The family Xenodermidae Gray, 1849 is a group of caenophidian snakes widely distributed in South, East, and Southeast Asia and including five recognized genera and 36 species (Deepak et al. 2021). Among them, *Achalinus* Peters, 1869 has the most species in this family, with 28 recognized species. Due to their cryptic lifestyle, small body size, and inconspicuous body color, they are hard to detect in the wild (Zhao et al. 1998; Zhao 2006; Ziegler et al. 2019). At present, there have been no cases of albinism in any species of Xenodermidae. *Achalinussheni* was first described from Loudi City and Nanyue District, Hunan Province, China in 2023 based on five male specimens (Ma et al. 2023a). During a recent herpetological survey in Yangshi Town, Hunan Province, China in July 2023, we collected one male and three female specimens of *A.sheni*. However, one of the adult females displayed a distinct pastel-yellow body coloration and red eyes, which is considered to be a completely albino individual. In addition, the newly collected female specimens also showed certain sex differences in morphology. Herein, we first report details of the specimen displaying albinism, provide photographs of the first female specimens of *A.sheni*, and present an extended diagnosis of this species.

## ﻿Materials and methods

### ﻿Morphometrics

Four snake specimens were collected from Yangshi Town, Lianyuan City, Hunan Province, China (specimen vouchers LFR2023008–LFR2023010 and LFR2024015). Specimens were humanely euthanized using lethal injection with 0.7% tricaine methanesulfonate (MS222) solution, and liver tissues were taken and preserved in 95% alcohol. Then the specimens were directly preserved in 75% ethanol and deposited in Qinghai University Museum. Sampling procedures involving live snakes were in accordance with the Wild Animals Protection Law of China and approved by the Institutional Ethics Committee of Qinghai University (protocol code SL-2023028). The sex of all specimens was determined by tail dissection.

Measurements and scale counts followed Zhao (2006) and Ma et al. (2023a). Three measurement characters were measured with Deli Stainless Ruler (No. 8462) to the nearest 1 mm : snout–vent length (**SVL**) ,tail length (**TAL**) and total length (**TL**). All other measurements were performed using Deli digital calipers (DL312200) to the nearest 0.1 mm : loreal height (**LorH**): measured from the highest part to the lowest part of the loreal in lateral view ; loreal length (**LorL**): measured from the most anterior loreal to the most posterior loreal in lateral view ; length of the suture between internasals (**LSBI**) ; length of the suture between prefrontals (**LSBP**) ; head length (**HL**): taken from the tip of snout to the posterior margin of mandible ; head width (**HW**): measured around the widest part of the head in dorsal view ; eye diameter (**ED**): taken from the most anterior corner of the eye to the most posterior corner ; length of supraocular (**SPOL**): horizontal distance between anterior and posterior tip of supraocular , and length of upper anterior temporal (**ATUL**): horizontal distance between anterior and posterior tip of upper anterior temporal. The scale characters and their abbreviations are as follows : supralabials (**SL**) ; infralabials (**IL**) ; infralabials touching the first pair of chin shields (**IL-1^st^ Chin**) ; loreals (**Lor**) ; preoculars (**PRO**) ; postoculars (**PO**) ; temporals (**TEMP**) ; supraoculars (**SPO**) ; dorsal scale rows (**DSR**) (counted at one-head-length behind the head, at midbody, at one-head-length before the cloacal plate ); ventral scales (**VS**) , cloacal plate (**CP**) , and subcaudals (**SC**).

### ﻿Molecular phylogenetic analyses

Genomic DNA was extracted from liver tissue using a Qiagin DNEasy Blood and Tissue Extraction Kit (Qiagen Inc., Valencia, CA). The partial mitochondrial DNA gene encoding cytochrome c oxidase subunit 1 (*CO1*) was obtained by polymerase chain reaction (PCR) using primer Chmf4 (5′-TYT CWA CWA AYC AYA AAG AYA TCG G-3′) and Chmr4 (5′-ACY TCR GGR TGR CCR AAR AAT CA-3′) (Che et al. 2012). PCR products were sequenced by Shanghai Map Biotech Co., Ltd. The raw sequences were stitched using SeqMan in the DNASTAR software package (Burland 2000) and the newly generated sequences were submitted to GenBank (Table 1).

**Table 1.** Localities, voucher information, GenBank numbers and references for all samples used in this study.

NO.	Species name	Locality	Voucher NO.	Genbank No.	References
1	*Achalinussheni*	Lianyuan, Hunan, China	LFR2023008	PP725554	This study
2	*A.sheni*	Lianyuan, Hunan, China	LFR2023009	PP725555	This study
3	*A.sheni*	Lianyuan, Hunan, China	LFR2023010	PP725556	This study
4	*A.sheni*	Lianyuan, Hunan, China	LFR2024015	PP725559	This study
5	*A.sheni*	Lianyuan, Hunan, China	ANU20230012	OR178145	Ma et al. 2023a
6	*A.sheni*	Lianyuan, Hunan, China	ANU20230013	OR178146	Ma et al. 2023a
7	*A.yunkaiensis*	Dawuling Forestry Station, Guangdong, China	SYS r001443	MN380329	Wang et al. 2019
8	*A.yunkaiensis*	Dawuling Forestry Station, Guangdong, China	SYS r001502	MN380330	Wang et al. 2019
9	*A.yunkaiensis*	Maoershan Nature Reserve, Guangxi, China	YBU 14612	MT365525	Yu et al. 2020
10	*A.yunkaiensis*	Xinning, Hunan, China	CIB 119041	OQ978852	Ma et al. 2023b
11	*A.ater*	Huaping Nature Reserve, Guangxi, China	SYS r00852	MN380334	Wang et al. 2019
12	*A.dabieshanensis*	Yaoluoping Nature Reserve, Anhui, China	AHU2018EE0710	MW316598	Zhang et al. 2023
13	*A.damingensis*	Nanning, Guangxi, China	ANU20220009	OP644487	Yang et al. 2023
14	*A.dehuaensis*	Dehua, Fujian, China	YBU 13013	MZ442662	Li et al. 2021
15	*A.emilyae*	Hoanh Bo, Vietnam	IEBR 4465	MK330857	Ziegler et al. 2019
16	*A.formosanus*	Taiwan, China	RN2002	KU529452	Unpublished
17	*A.hunanensis*	Huaihua, Hunan, China	CIB 119039	OQ848425	Ma et al. 2023c
18	*A.huangjietangi*	Huangshan, Anhui, China	HSR18030	MT380191	Huang et al. 2020
19	*A.juliani*	Ha Lang, Cao Bang, Vietnam	IEBR A.2018.8	MK330854	Ziegler et al. 2019
20	*A.meiguensis*	Mianyang, Sichuan, China	GP835	MZ442641	Li et al, 2021
21	*A.nanshanensis*	Huaihua, Hunan Province, China	HNNU230901	OR523368	Li et al. 2024
22	*A.niger*	Taiwan, China	RN0667	KU529433	Unpublished
23	*A.ningshanensis*	Ningshan, Shaanxi, China	ANU 20220006	ON548422	Yang et al. 2022
24	*A.panzhihuaensis*	Yanbian, Sichuan, China	KIZ 040189	MW664862	Hou et al. 2021
25	*A.pingbianensis*	Honghe, Yunnan, China	YBU 18273	MT365521	Li et al. 2020b
26	*A.quangi*	Phu Yen, Son La, Vietnam	ZVNU.2022.08	OQ197471	Pham et al. 2023
27	*A.rufescens*	Hongkong, China	SYS r001866	MN380339	Wang et al. 2019
28	*A.* sp1	Ningshan, Shaanxi, China	LFR2023038	PP725557	This study
29	*A.* sp1	Ningshan, Shaanxi, China	LFR2023039	PP725558	This study
30	*A.* sp2	Taibai, Shaanxi, China	CHS007	MK064591	Li et al. 2020a
31	*A.spinalis*	Badagong Mountains, Hunan, China	SYS r001327	MN380340	Wang et al. 2019
32	*A.timi*	Thuan Chau, Son La, Vietnam	IEBR A.2018.10	MK330856	Ziegler et al. 2019
33	*A.tranganensis*	Ninh Binh, Vietnam	VNUF R.2018.21	MW023086	Luu et al. 2020
34	*A.vanhoensis*	Van Ho, Son La, Vietnam	VNUF R.2019.13	ON677935	Ha et al. 2022
35	*A.yangdatongi*	Wenshan Nature Reserve, Yunnan, China	KIZ 034327	MW664865	Hou et al. 2021
36	*A.zugorum*	Bac Me, Ha Giang, Vietnam	IEBR 4698	MT502775	Miller et al. 2020
	**Out group**				
37	*Fimbriosklossi*	Quang Ngai, Vietnam	IEBR 3275	KP410744	Teynié et al. 2015
38	*Parafimbrioslao*	Louangphabang, Laos	MNHN 2013.1002	KP410746	Teynié et al. 2015
39	*Stoliczkiavanhnuailianai*	Mizoram, India	BNHS 3656	OL422476	Deepak et al. 2021

Except for the newly generated sequence, 32 sequences of 25 recognized species and one unnamed of genus *Achalinus*, and three outgroups (selected as Ma et al. 2023a): *Fimbriosklossi* Smith, 1921, *Parafimbrioslao* Teynié, David, Lottier, Le, Vidal & Nguyen, 2015, and *Stoliczkiavanhnuailianai* Lalronunga, Lalhmangaiha, Zosangliana, Lalhmingliani, Gower, Das & Deepak, 2021 were downloaded from the National Center for Biotechnology Information (NCBI) (Accession numbers listed in Table 1). The *CO1* sequences (624 bp) were input in MEGA X software (Kumar et al. 2018) and aligned by MUSCLE (Edgar 2004). Maximum likelihood (ML) was used to infer tree structure with IQ-TREE v. 1.6.12 (Nguyen et al. 2015). The best-fit model, TN+F+I+G4, was inferred using a Bayesian Information Criterion (BIC) with the program ModelFinder (Kalyaanamoorthy et al. 2017). Ultrafast Bootstrap Approximation (UFB) node support was assessed by using 5000 ultrafast bootstrap replicates, and SH-like approximate likelihood ratio test (SH-aLRT) was conducted to the single branch tests by 1000 replicates. In addition, we calculated the uncorrected pairwise distances (*p*-distances) using the MEGA X software (Kumar et al. 2018).

## ﻿Results

**Figure 1.**
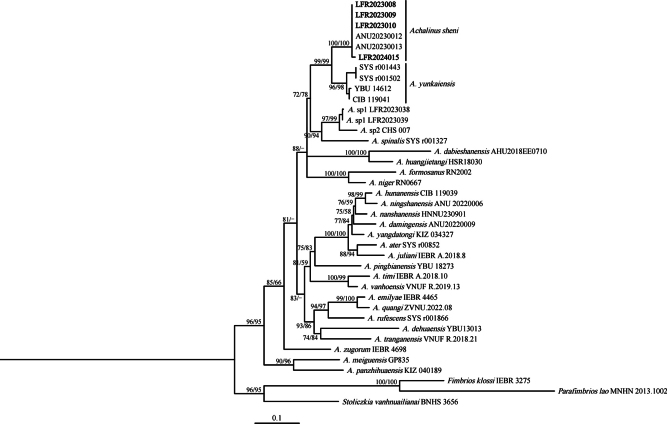
Maximum-likelihood tree of the genus *Achalinus* inferred from the *CO1* gene fragment. The nodes supporting values on branches are presented as SH-like approximate likelihood ratio test (SH) / Ultrafast Bootstrap Approximation (UFB), the ones lower than 50 are displayed as “–”.

The phylogeny inferred using the mitochondrial fragment *CO1* (624 bp) demonstrated that the newly collected specimens from Yangshi Town, Hunan Province, China were clustered together with selected *A.sheni* type series (SH 100 / UFB 100) (Fig. 1). We also show that intraspecific generic divergence ranged from 0.0%–0.8% was detected (Table 2), which is less than the minimum interspecific uncorrected *p*-distance among other recognized species of *Achalinus*, indicating that the newly collected *Achalinus* specimens should be identified as *A.sheni*.

**Table 2.** Uncorrected *p*-distances (%) among the *Achalinus* species based on partial mitochondrial *CO1* gene for species compared in this study.

ID	Species	1–6	7	8	9	10	11	12	13	14	15	16	17	18	19–20	21	22	23	24	25	26	27	28	29	30	31	32–35
1–6	*A.sheni*	0–0.8																									
7	*A.ater*	13.1–13.4	–																								
8	*A.dabieshanensis*	15.3–15.9	14.8	–																							
9	*A.damingensis*	13.3–13.6	7.4	15.9	–																						
10	*A.dehuaensis*	13.8	16.1	18.6	15.2	–																					
11	*A.emilyae*	13.6–14.2	11.2	18.0	12.9	15.3	–																				
12	*A.formosanus*	12.7–12.9	13.3	18.8	14.2	15.7	13.6	–																			
13	*A.huangjietangi*	12.9	13.1	11.0	15.2	15.3	15.5	16.1	–																		
14	*A.hunanensis*	12.7–13.1	7.6	17.0	5.7	15.3	13.8	13.6	15.0	–																	
15	*A.juliani*	13.6–14.0	6.6	15.9	8.3	14.8	12.9	11.4	14.4	9.1	–																
16	*A.meiguensis*	14.2–14.8	15.3	18.0	16.5	18.4	15.3	15.5	16.9	16.3	16.7	–															
17	*A.niger*	12.9–13.1	12.9	16.1	13.3	16.3	12.7	8.5	15.7	13.3	11.7	13.8	–														
18	*A.ningshanensis*	13.6–14.0	7.4	17.2	7.4	15.5	13.8	14.2	15.5	3.4	9.5	16.9	14.0	–													
19–20	*A.* sp1	9.8–10.4	12.7–12.9	14.4–14.6	11.9–12.1	13.8–14.0	12.9–13.1	12.7–12.9	13.6–13.8	13.1–13.3	12.1–12.3	15.0–15.2	9.8–10.0	13.8–14.0	0.2												
21	*A.nanshanensis*	13.6–14.0	6.8	16.1	5.1	13.4	13.3	13.6	14.6	4.9	8.1	17.6	12.1	5.7	12.5–12.7	–											
22	*A.panzhihuaensis*	14.8	16.5	16.5	15.5	15.5	16.5	16.1	15.7	16.5	15.7	11.4	14.0	17.4	14.4–14.6	15.3	–										
23	*A.pingbianensis*	11.2	11.0	15.3	10.2	14.6	13.1	14.2	14.0	11.0	11.6	16.7	11.9	11.6	10.0–10.2	11.0	14.8	–									
24	*A.quangi*	14.2–14.8	11.4	18.4	12.7	15.5	2.8	13.6	15.9	13.6	12.5	15.2	12.1	13.1	12.3–12.5	12.7	16.9	13.6	–								
25	*A.rufescens*	13.1	11.7	15.9	12.1	12.9	9.7	13.8	14.6	11.7	11.2	18.6	13.8	11.9	13.4–13.6	11.4	15.9	12.7	10.0	–							
26	*A.spinalis*	11.7–12.3	14.6	16.5	14.6	14.2	14.4	14.2	14.4	14.0	14.0	15.9	13.8	15.2	8.9–9.1	14.4	16.1	13.3	13.6	12.7	–						
27	*A.* sp2	11.0	14.0	14.8	13.4	15.3	13.3	14.2	13.6	15.0	13.8	15.7	11.9	15.7	3.2–3.4	13.8	15.5	12.3	13.1	14.8	10.4	–					
28	*A.timi*	13.3–13.6	12.7	16.5	12.5	15.0	12.9	13.3	15.9	12.1	13.4	15.9	11.6	12.9	11.4–11.6	13.1	15.3	11.9	12.5	14.0	14.0	12.9	–				
29	*A.tranganensis*	14.2–14.6	12.5	15.3	13.8	14.0	12.3	16.9	13.4	14.8	14.2	16.3	14.6	15.0	13.1–13.3	13.4	16.5	13.4	11.7	12.7	15.5	13.6	13.4	–			
30	*A.vanhoensis*	12.7–13.1	11.9	15.5	11.7	14.8	11.7	13.6	15.2	11.4	12.7	15.7	11.7	11.7	11.2–11.4	11.9	15.3	10.6	11.6	12.9	12.3	12.7	4.5	11.9	–		
31	*A.yangdatongi*	14.0–14.4	6.4	16.7	5.7	14.4	12.7	14.2	14.8	5.1	7.6	17.2	13.4	5.9	12.1–12.3	4.5	15.7	10.8	12.5	11.6	14.2	13.6	12.7	12.9	10.8	–	
32–35	*A.yunkaiensis*	6.4–7.2	11.9–12.9	15.0–15.9	12.3–12.9	14.4–14.8	12.7–13.1	11.9–12.5	14.0–14.2	11.7–12.3	12.3–12.9	15.3–15.9	10.4–11.9	12.7–13.3	9.3–10.0	11.6–12.5	15.7–16.1	10.8–11.4	12.7–13.6	12.1–13.4	11.7–11.9	10.0–11.0	12.7–13.3	13.1–14.0	11.7–12.3	12.3–12.5	0.0–3.0
36	*A.zugorum*	10.4	13.3	15.3	12.3	14.2	12.9	13.4	15.0	12.1	13.3	15.0	13.1	12.5	11.6–11.7	12.7	15.2	10.2	13.1	13.8	13.4	13.6	13.4	11.7	11.7	12.1	10.4–11.9

### ﻿Taxonomic account

**Reptilia**

**Serpentes**

**Xenodermidae**

***Achalinus***

#### ﻿ Achalinus sheni

Taxon classificationAnimaliaSquamataXenodermidae

Ma, Xu, Qi, Wang, Tang, Huang & Jiang, 2023

6B1972E5-435B-5ADF-B4E1-C85CB127250F

**Figure 2.**
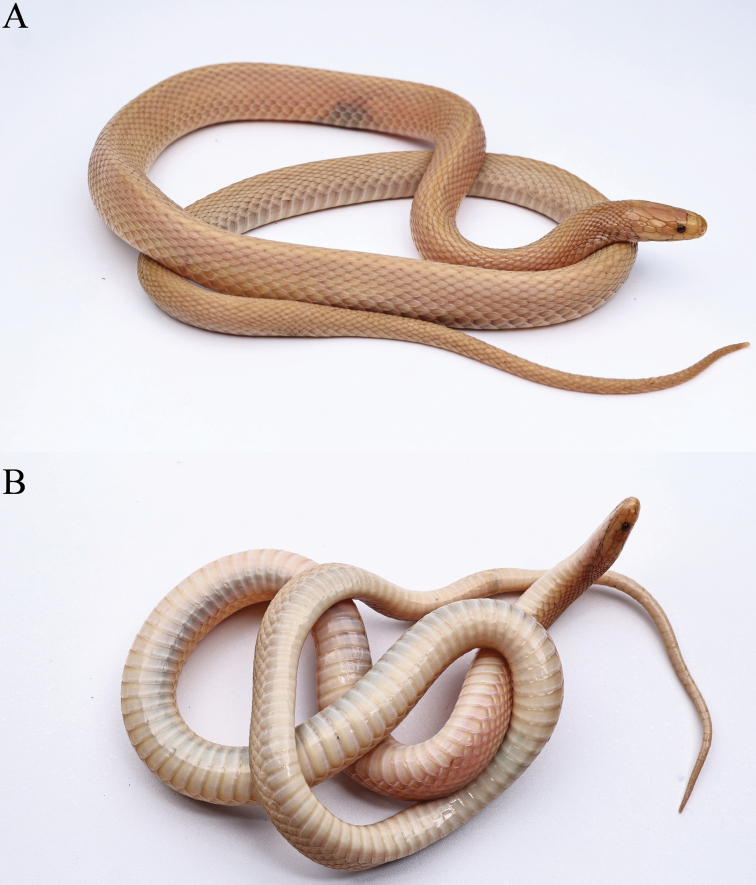
Adult female albinistic specimen of *Achalinussheni* in life (LFR2024015) **A** dorsal view **B** ventral view. Photos by Yu-Hao Xu.

**Figure 3.**
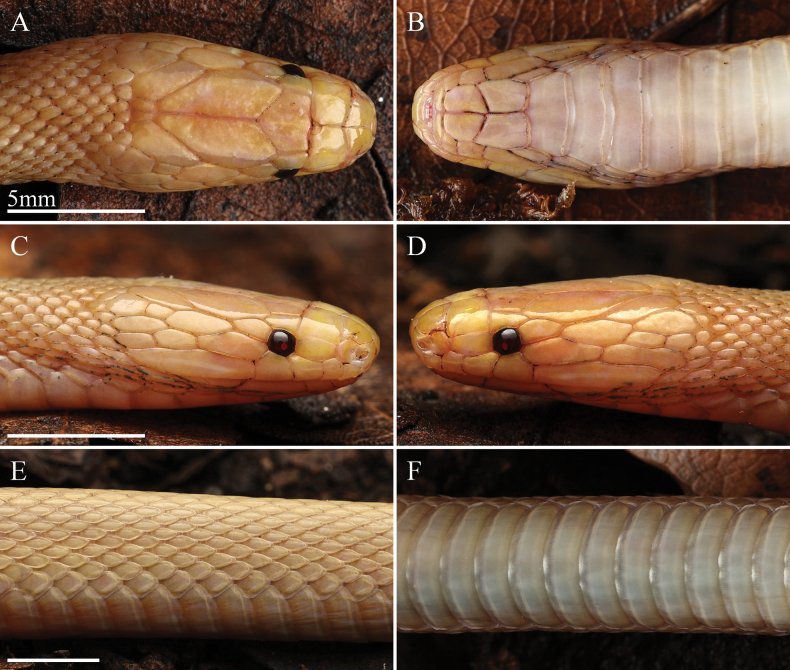
Close-up view of the adult female albinistic specimen of *Achalinussheni* in life (LFR2024015) **A** dorsal view of the head **B** ventral view of the head **C** right view of the head **D** left view of the head **E** lateral view of the middle body **F** venter view of the middle body. Photos by Yu-Hao Xu.

**Figure 4.**
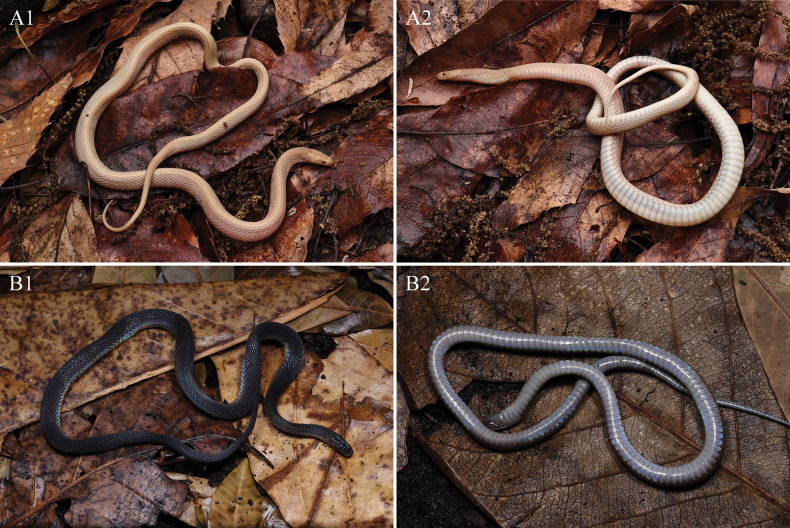
Comparisons between the albinistic specimen and typical specimen of *Achalinussheni* in life **A1–A2** LFR2024015 **B1–B2** LFR2023010 **A1**, **B1** dorsal view **A2**, **B2** ventral view. Photos by Yu-Hao Xu.

**Figure 5.**
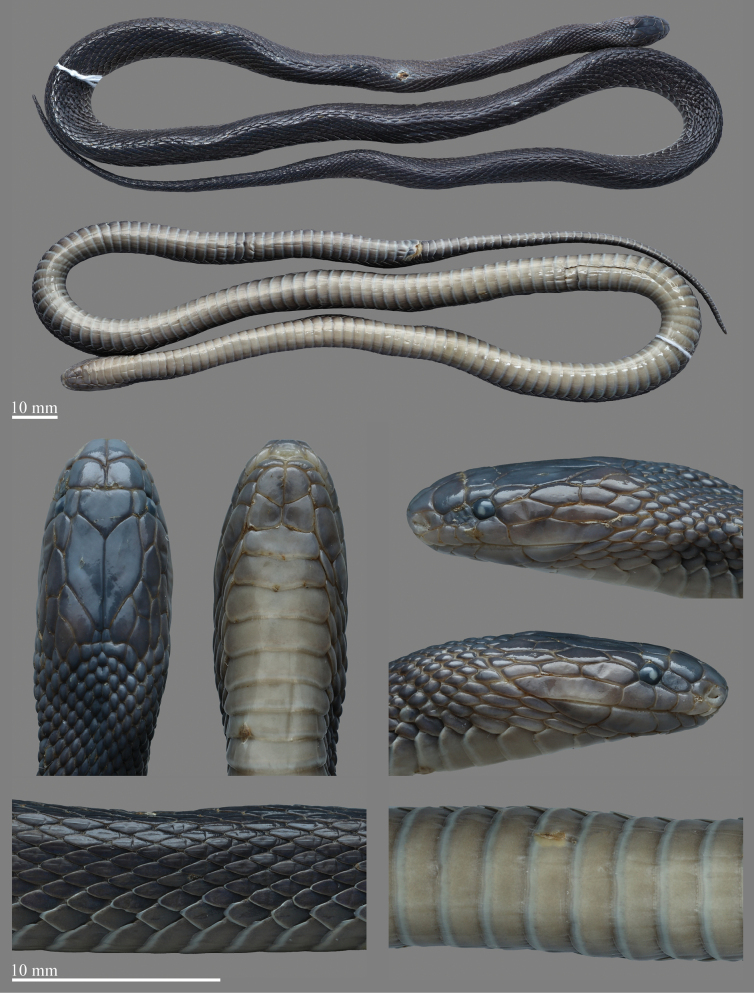
Preserved specimen of the typical female specimen of *A.sheni* (LFR2023008). Photos by Yu-Hao Xu.

**Figure 6.**
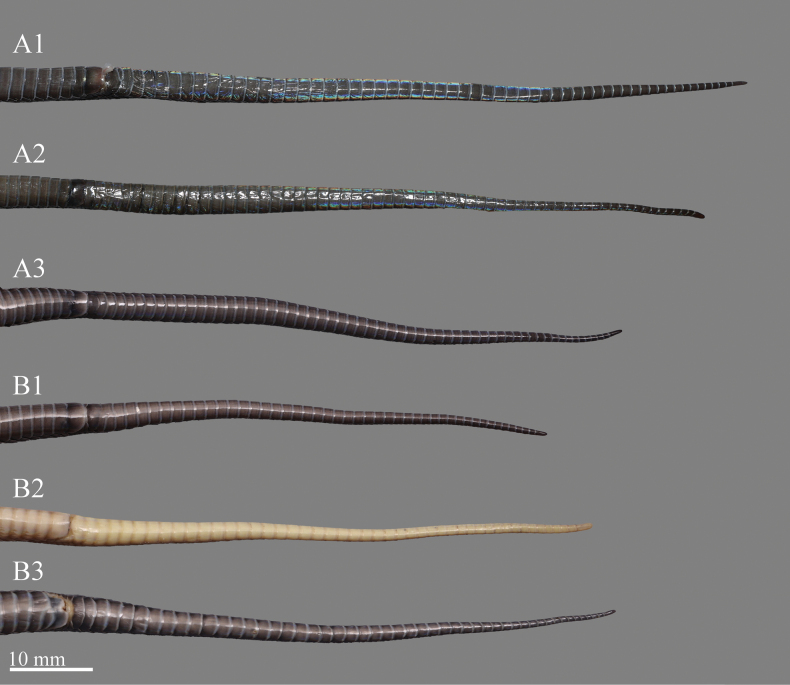
Comparisons of the tails between males and females of *Achalinussheni***A1** specimen ANU20230013, paratype, adult male **A2** specimen ANU20230012, paratype, adult male **A3** specimen LFR2023009, adult male **B1** specimen LFR2023010, adult female **B2** specimen LFR2024015, adult female **B3** specimen LFR2023008, adult female. Photos by Yu-Hao Xu.

Figs 2 , 3 , 4 , 5 , 6 Common name: Shen’s Odd-scale Snake / Shěn Shì Jǐ Shé (沈氏脊蛇)

##### Specimens examined.

Three typical specimens: LFR2023008 (adult female), LFR2023009 (adult male), LFR2023010 (adult female); and one albinistic specimen: LFR2024015 (adult female), collected in July, 2023 from Yangshi Town, Lianyuan City, Loudi City, Hunan Province (27°32'07.08"N, 111°48'31.68"W, 370 m a. s. l.) ; coll. by Shu Li and Ziyuan Feng.

##### Description of the albinistic specimen.

***Measurements and scalation*.** An adult female specimen (field number LFR2024015) with SVL 354 mm (TL 416 mm and TAL 62 mm); tail relatively short, TAl/TL ratio 0.149; body slender and cylindrical; head slightly distinct from the neck; HW 5.8 mm; HL 10.8 mm; eye small; ED 1.1 mm; rostrum small, triangular, slightly visible from above; length of the suture between the internasals (LSBI 1.25 mm) subequal to the length of the suture between the prefrontals (LSBP 1.32 mm), LSBI/LSBP ratio 0.95; nostril in the anterior part of the nasal; prefrontals paired; frontal single, pentagonal, pointing to the rear, the width and length close; loreal one, subrectangular, LorL 1.6 mm, LorH 0.9 mm, LorH/LorL ratio 0.56; supraocular one, pentagonal, SPOL 1.9 mm; TEMP 7/8, arranged in three rows (2+1+4 in left and 2+2+4 in right), the anterior two contact the eye, ATUL 1.7 mm, SPOL/ATUL ratio 1.1; SL 6, the 4^th^–5^th^ contact the eye, the last one much elongated; two pairs of chin shields, the anterior pairs longer than the posterior pairs, followed by preventrals; one mental; IL 5, the first one contact with each other after the mental and before the 1^st^ chin-shields, 1^st^–3^rd^ touch the first pair of chin-shields.

Dorsal scales strongly keeled, lanceolate, 23 rows throughout the body, the outmost row smooth and significantly enlarged. VS 164; anal entire; SC 46, not paired.

***Coloration*.** The comparison of color pattern between the albinistic specimen and the typical specimen are shown in Fig. 4. In life, dorsum (head, body, and tail) predominantly pastel yellow or paster orange owning to the lack of melanophoric pigments, and the iridescence on the body surface also disappears. Head scales in dorsal view same as dorsum, interstitial skin of dorsal and sutures of head scales milk star white. The iris was blood-red, with a reddish pupil. Supralabials, mental, and infralabials were pastel orange. The ventral ground color of body and tail were milk star white, darker on both sides than in the middle, and with free margins of ventral scales and subcaudals almost transparent with a slight hint of pastel orange (Figs 2, 3).

##### Expanded description of the females.

Measurements and scalation data of the newly collected specimens (1 male and 3 females) are presented in Table 3. Based on three newly collected female specimens (field number: LFR2023008, LFR2023010, LFR2024015), an expanded description of the females is provided as below.

**Table 3.** Morphological variation characters in the newly collected *Achalinussheni* specimens.

Voucher number	LFR2023008	LFR2023009	LFR2023010	LFR2024015
**Sex**	♀	♂	♀	♀
**SVL**	341	257	298	354
**TL**	408	324	355	416
**TAL**	67	67	57	62
**TAL/TL**	0.164	0.207	0.160	0.149
**HL**	11.4	9.6	11.5	10.8
**HW**	6.6	5.1	5.5	5.8
**SL**	3+2+1	3+2+1	3+2+1	3+2+1
**IL**	5/5	5/5	5/5	5/5
**Chin**	2	2	2	2
**IFL–1stChin**	1^th^–3^rd^	1^th^–3^rd^	1^th^–3^rd^	1^th^–3^rd^
**Lor**	1	1	1	1
**LorH**	1.2	1.0	1.3	0.9
**LorL**	1.7	1.6	1.7	1.6
**LorH/LorL**	0.71	0.63	0.76	0.56
**LSBI**	1.8	1.3	1.5	1.23
**LSBP**	1.6	1.1	1.6	1.32
**LSBI/LSBP**	=	=	=	=
**ED**	1.1	0.9	1.1	1.1
**PrO**	0	0	0	0
**PO**	0	0	0	0
**TEMP**	2+2+3/2+2+3	2+2+3/2+2+4	2+2+3/2+2+3	2+1+4/2+2+4
**ATUL**	1.7	1.3	1.7	1.7
**SPO**	1	1	1	1
**SPOL**	1.6	1.3	1.6	1.9
**SPOL/ATUL**	0.94	1.00	0.94	1.12
**DSR**	23-23-23	23-23-23	23-23-23	23-23-23
**VS**	172	167	172	174
**CP**	entire	entire	entire	entire
**SC**	49	59	47	46

***Measurements and scalation*.** Tail relatively short, TAL/TL ratio 0.149–0.164; body slender and cylindrical, the maximal TL 416 mm with SVL 354 mm and TAL 62 mm; head relatively narrow, slightly distinct from the neck, HL 10.8–11.5 mm; HW 5.5–6.6 mm; rostrum small, triangular, slightly visible from above; eye small, pupil round, ED 1.1 mm; LSBI subequal to LSBP; nostril in the anterior part of the nasal; prefrontals 2, elongated; frontal 1, pentagonal, pointing to the rear, the width and length close; loreal one, subrectangular, LorL 1.6–1.7 mm, LorH 0.9–1.3 mm, LorH/LorL ratio 0.56–0.76; supraocular one, pentagonal, SPOL 1.6–1.9 mm; temporals long, arranged in three rows, TEMP 2+1+4, 2+2+3 or 2+2+4, the anterior two contact the eye, ATUL 1.7 mm, SPOL/ATUL ratio 0.94–1.12; SL 6, the 4^th^–5^th^ contact the eye, the last one much elongated; two pairs of chin shields, the anterior pairs longer than the posterior pairs, followed by preventrals; one mental; IL 5, the first one contact with each other after the mental and before the 1^st^ chin-shields, 1^st^–3^rd^ touch the first pair of chin-shields.

Dorsal scales 23-23-23, lanceolate and strongly keeled, the outmost row smooth and significantly enlarged. VS 172–174; CP entire; SC 46–49, unpaired.

***Coloration in life*.** In life, the dorsum (head, body, and tail) is predominantly brownish black and slightly tinged with iridescence. Head scales in dorsal view are the same as the dorsum, and with the middle darker than the sides. Dorsum brownish black and the five innermost dorsal scale rows a little darker, forming an inconspicuous longitudinal vertebral line. Eyes pure black. Mental, infralabials, and chin shields light grayish brown. Ventral ground color of body and tail generally light grey or light taupe and darker on the sides. The free margins of ventral scales are greyish white (Fig. 4).

***Coloration in preservation*.** In preservation, coloration still resembles the specimen in life, except that the coloration of dorsum further deepening, and the background color of the venter becomes light brownish grey (Fig. 5).

##### Variation.

The female specimens have a similar color pattern as male specimens, but in measurement and scalation features, there is variation by sex: females have a relatively large body size (TL 355–408 mm vs 149–371 mm in male); a significantly short tail, TAL/TL ratio 0.149–0.164 (vs 0.183–0.224 in male) (Fig. 6); more ventral scales (172–174 vs 161–170 in male); and fewer subcaudals (46–49 vs 55–61 in male) (Table 4).

**Table 4.** Comparison of the key morphological characters between *Achalinussheni* and *A.yunkaiensis* obtained from specimens examined in this study, Wang et al. (2019), and Ma et al. (2023a).

Sex	*A.sheni*		*A.yunkaiensis*	
	♂	♀	♂	♀
**N**	6	3	4	3
**SVL**	122–292	298–354	189–359	204–386
**TL**	149–371	355–416	232–418	256–488(+)
**TAL**	27–80	57–67	43–63	52–73
**TAL/TL**	**0.183–0.224**	**0.149–0.164**	**0.185–0.200**	**0.156–0.204**
**SL**	3+2+1	3+2+1	3+2+1	3+2+1
**IL**	**5 (rarely 6)**	**5**	**6**	**6**
**Chin**	2	2	2	2
**IFL–1stChin**	1^th^–3^rd^	1^th^–3^rd^	1^th^–3^rd^	1^th^–3^rd^
**Lor**	1	1	1	1
**LorH**	0.7–1	0.9–1.3	0.8–1.3	0.7–1.2
**LorL**	1.3–1.7	1.6–1.7	1.3–2.2	1.5–2.2
**LorH/LorL**	0.53–0.93	0.56–0.76	0.56–0.64	0.49–0.55
**LSBI vs LSBP**	=	=	=	=
**TEMP**	2+2+3	2+2+3 or 2+2+4 or 2+1+4	2+2+3 or 2+2+4	2+2+3 or 2+2+4
**ATUL**	1.3–1.5	1.7	1.2–2.2	1.9–2.9
**SPO**	1	1	1	1
**SPOL**	1.1–1.6	1.6–1.9	1–1.6	1.3–1.6
**SPOL/ATUL**	**0.99–1.16**	**0.94–1.12**	**0.66–0.83**	**0.55–0.65**
**DSR**	23-23-23	23-23-23	23-23-23	23-23-23
**VS**	**161–170**	**172–174**	**151–162**	**144–156**
**CP**	1	1	1	1
**SC**	**55–61**	**46–49**	**49–56**	**51–55**
**VS+SC**	**220–226**	**219–221**	**200–212**	**195–205**

##### Revision of diagnostic characters.

(1) dorsal scales strongly keeled, 23 rows throughout the body, the outmost row smooth and significantly enlarged; (2) tail relatively short, TAL/TL ratio 0.183–0.224 in males, and 0.140–0.164 in females; (3) the suture between internasals subequal to the suture between prefrontals; (4) loreal one, subrectangular, LorH/LorL 0.53–0.76; (5) ventrals 161–170 in males and 172–174 in females; (6) cloacal plate entire; (7) subcaudals 55–61 in males and 46–49 in females, not paired; (8) the length of supraocular almost equal to the length of upper anterior temporal; (9) vertebral line inconspicuous and subcaudal streak absent.

##### Natural history notes.

*Achalinussheni* is currently known from Hunan Province, China: Lianyuan City, Nanyue District and Nanshan National Park, Shaoyang City (350–410 m a.s.l.). The known activity period of *A.sheni* is from March to October but activity peaks in early summer. The species usually prefers to hide under rocks, decaying wood, or fallen leaves, but it has sometimes been found on cement roads in the mountains after rain or on high-humidity nights. Through dissection, it was found that there were undigested earthworms in the intestine of specimen LFR2023009. Therefore, we speculate that *A.sheni* feeds mainlyon worms in the wild.

## ﻿Discussion

The genus *Achalinus* is widely distributed in Vietnam, China, and Japan (Zhao et al. 1998; Zhao 2006), with 28 currently recognized species, and lately it has attracted much attention in scientific literature (Wang et al. 2019; Ziegler et al. 2019; Li et al. 2020b; Luu et al. 2020; Hou et al. 2021; Huang et al. 2021; Li et al. 2021; Ha et al. 2022; Yang et al. 2022; Ma et al. 2023a, 2023b, 2023c; Pham et al. 2023; Yang et al. 2023; Zhang et al. 2023; Li et al. 2024). However, no cases of albinism have been described to our knowledge. Therefore, this first report of albinism in *A.sheni* sheds light on this rare phenomenon in the genus and family.

Species of *Achalinus* typically exhibit a rainbow-colored iridescence on their body surface especially when exposed to sunlight or camera flash. However, when observing the albino individual, we found that the rainbow color on their body surface almost completely disappeared. It is currently unclear whether the lack of iridescence is entirely caused by the disappearance of melanin. In the future, we will further examine microstructure of albinism and examine the genetic underpinnings of this phenomenon.

In this study, we provide the first detailed description and photographs of the female of *A.sheni* and compare the morphological differences between males and females. We demonstrate intersexual differences such as the total length, the tail length, and the number of venter scales and subcaudals, which will help distinguish this species from other closely related species, especially its sister species *A.yunkaiensis* (Table 4), which is sympatric in distribution with *A.sheni* in the Nanshan National Park (Li et al. 2024).

Moreover, in this study, we provide two partial *CO1* sequences of two *Achalinus* specimens from Ningshan County, Shaanxi Province, China, which cluster with the Taibai specimen (considered as *Achalinus* sp. by Yang et al. (2023)) (Table 1) with high support values (SH 97 / UFB 99) (Fig. 1). Interestingly, the uncorrected *p*-distance was 3.2–3.4%, indicating substantial genetic differences between the two populations, but further population genomic investigation is needed to properly understand biogeographic causes of this putative population structure. However, the morphological examination indicated that these two specimens from Ningshan County are consistent with the original descriptions of *A.ningshanensis*. Therefore, broad sampling of morphological and genomic data is required to better understand population or species structuring within *A.ningshanensis*.

## Supplementary Material

XML Treatment for Achalinus sheni

## ﻿Additional information

### Author ORCIDs

Yu-Hao Xu https://orcid.org/0000-0001-6094-6680

Li-Fang Peng https://orcid.org/0000-0001-9325-7048

### Data availability

All of the data that support the findings of this study are available in the main text.
